# Identification of a novel *AGO2* variant causing LESKRES in a Chinese family with intellectual disability

**DOI:** 10.3389/fgene.2025.1598462

**Published:** 2025-06-12

**Authors:** Shufa Yang, Wei Song, Yousheng Yan

**Affiliations:** ^1^ Prenatal Diagnostic Center, Beijing Obstetrics and Gynecology Hospital, Beijing Maternal and Child Healthcare Hospital, Capital Medical University, Beijing, China; ^2^ Department of Obstetrics, Beijing Obstetrics and Gynecology Hospital, Beijing Maternal and Child Healthcare Hospital, Capital Medical University, Beijing, China

**Keywords:** Lessel-Kreienkamp syndrome, *AGO2*, whole-exome sequencing, molecular dynamics analysis, intellectual disabilities

## Abstract

**Background:**

Lessel-Kreienkamp syndrome (LESKRES, MIM #619149), an autosomal dominant genetic disorder caused by variants in *AGO2* (MIM*606229), primarily leads to neurodevelopmental symptoms.

**Objective:**

This study aims to investigate the genetic etiology of a family with intellectual disability.

**Methods:**

Whole-exome sequencing (WES) was used to initially identify the pathogenic variants responsible for the intellectual disability in the family, and Sanger sequencing was employed for confirmation. Complete family information was collected, and Sanger sequencing was performed to confirm the co-segregation of the variant with the intellectual disability, thereby determining the pathogenicity of the novel variant. The pathogenicity of the novel variant was evaluated using *in silico* methods.

**Results:**

All four intellectual disability individuals carried the novel *AGO2* (NM_012154.5): c.2149T>C (p.Cys717Arg) variant, while the other individuals did not. According to ACMG guidelines, this novel variant is classified as likely pathogenic. The novel variant occurs at a conserved position in *AGO2* and is predicted to affect the 3D structure of the *AGO2* protein.

**Conclusion:**

This study identifies a novel *AGO2* variant causing LESKRES in the Chinese population for the first time. Our findings expand the variants spectrum of *AGO2* leading to LESKRES and highlight the value of WES in diagnosing genetic causes of intellectual disabilities.

## 1 Introduction

Lessel-Kreienkamp syndrome (LESKRES, MIM #619149) is a neurodevelopmental disorder marked by global developmental delay, intellectual disability, and delays in speech and language development, which are noticeable from infancy or early childhood. The severity of the condition varies widely: some individuals experience mild delays in walking and minor cognitive impairments, while others may be non-ambulatory and nonverbal. Behavioral issues are common, and additional characteristics, such as seizures, hypotonia, abnormal gait, vision problems, heart defects, and subtle dysmorphic facial features, can also occur (Lessel et al., 2020). Lessel et al. reported that the 21 cases of LESKRES were caused by variants in the argonaute 2 (*AGO2*). The inheritance pattern of LESKRES syndrome is autosomal dominant. However, the majority of heterozygous variants found in LESKRES patients were *de novo* (Lessel et al., 2020).

*AGO2* (MIM*606229), located at chromosome 8q24.3, and spanning a 122 kb genomic region, belongs to AGO proteins. Humans have four types of AGO proteins (*AGO1*, *AGO2*, *AGO3*, and *AGO4*), which can bind to miRNA and siRNA. *AGO2*, also known as *EIF2C2* or *CASC7*, is a key component of the RNA-induced silencing complex (RISC), involved in both mRNA inhibition and degradation (Nakanishi, 2022). MicroRNA (miRNA) precursors are transcribed, processed into mature miRNAs, and then incorporated into Argonaute (*AGO1-4*) proteins to form RISC complex (Treiber et al., 2019). Polymorphisms in the *AGO2* gene are associated with various clinical conditions, including lymph node metastasis of nasopharyngeal carcinoma, progression of renal cell carcinoma, progression of prostate cancer, alcohol dependence, depression risk, and recurrent miscarriage (Teixeira et al., 2021; Li et al., 2015; Nikolic et al., 2017; Gedik et al., 2015; Kowalczyk et al., 2022; Kim et al., 2019). So far, approximately 13 pathogenic variants of various types in *AGO2* associated with LESKRES have been reported.

In the current study, we report a LESKRES family and perform genetic and clinical examinations on the family members. A novel variant in *AGO2* was identified. In-silico analysis, including interspecies conservation and molecular dynamics simulation was carried out to predict the pathogenicity of the novel missense variant. Our findings will aid in establishing the link between different *AGO2* variants and their associated phenotypes.

## 2 Materials and methods

### 2.1 Subjects and clinical evaluation

In April 2024, a 22-year-old woman with intellectual disability visited the genetic counseling clinic at our hospital, accompanied by her family. We conducted an extensive clinical survey and then collected peripheral blood samples from the family members in the pedigree for genetic testing.

### 2.2 Genetic testing

The extracted peripheral blood DNA was digested, fragmented, and end-repaired. The exons and adjacent splicing regions of the genes were captured using Amcare probes (AmCare Genomics Lab, Guangzhou, China). A DNA library was then prepared using the Gene Sequencing Library Kit (AmCare Genomics Lab, Guangzhou, China) and subjected to high-throughput sequencing on the AmCareSeq-2000 sequencer (AmCare Genomics Lab, Guangzhou, China).

The obtained next-generation sequencing (NGS) data were processed sequentially with the fastp, BWA, GATK, and ANNOVAR for data cleaning, alignment to the hg19 genome, variant calling, and variant annotation. Variant analysis and interpretation incorporated pathogenic variant databases (ClinVar, HGMD, DECIPHER, ISCA, and NCBI), population databases (gnomAD, ExAC), and the OMIM database. Variant filtering was performed using the following criteria: (1) Low-quality (read depth <20×, an allele fraction <30%). (2) Variants with a minor allele frequency ≥0.005, based on gnomAD and ExAC. (3) Variants located outside of coding regions and splicing regions (defined as the eight bases flanking each exon–intron boundary) were excluded. (4) Synonymous variants within the exome were removed. Given the clear autosomal dominant inheritance pattern revealed by pedigree analysis, we focused on variants inherited from the mother. The pathogenicity of variants was classified based on ACMG variant classification guidelines and supplemental guidelines, combined with clinical manifestations and examination results (Richards et al., 2015).

Exome sequencing results were validated using Sanger sequencing. Primers for candidate variants were designed with Primer Premier v5.0, followed by PCR amplification and purification. Sanger sequencing was performed on an ABI Prism 3700 sequencer.

### 2.3 Structural analysis

The 3D structure of *AGO2* was predicted and modeled using the AlphaFold program. The p.Cys717Arg and p. Gly733Arg variants was introduced into the wild-type *AGO2* protein model using Swiss-Pdb Viewer, followed by energy minimization. Molecular dynamics simulations were performed using the GROMACS (version 2020.6) (Rakhshani et al., 2019). The *AGO2*-WT, *AGO2*-(p.Cys717Arg), and *AGO2*-(p. Gly733Arg) model were simulated for 60 ns in the CHARMM36 force field (Soteras Gutierrez et al., 2016). The wild-type and variant protein structures were solvated in a triclinic water box, maintaining a minimum buffer distance of 1.0 nm between the protein surfaces and the edges of the simulation cell. The system was neutralized by adding Na^+^ and Cl^−^ ions to achieve charge balance. Subsequent energy minimization was conducted using the steepest descent algorithm to eliminate steric clashes and unfavorable geometries. Finally, the system underwent equilibration in the NVT ensemble at 300 K for 120 ns to ensure thermal and conformational stability prior to production simulations. After completing the molecular dynamics simulation, the following commands: gmx rms, gmx rmsf, gmx gyrate, gmx sasa, gmx hbond, and gmx do_dssp were used to calculate the following properties for both the wild-type and variant proteins: root-mean-square deviation (RMSD), root-mean-square fluctuation (RMSF), radius of gyration (Rg), solvent-accessible surface area (SASA), the number of intra-protein hydrogen bonds, and changes in secondary structure.

### 2.4 Analysis of missense variants

The evolutionary conservation of amino acid (AA) residues affected by specific missense variants was analyzed using UGENE (http://ugene.net/) with default parameters.

### 2.5 Ethics approval

This study was reviewed and approved in advance by the Ethics Committee of Beijing Obstetrics and Gynecology Hospital, Capital Medical University (approval No. 2025-KY-029-01). All procedures involving human participants adhered to the Declaration of Helsinki 1964 and its subsequent revisions, or other applicable ethical standards.

### 2.6 Consent to participate and to publish

All participants in this study were thoroughly informed about the study’s purpose, procedures, potential risks, and benefits. They were assured of their right to withdraw from the study at any time without facing any consequences. Written informed consent was obtained from all patients or their legal representatives, covering both genetic testing and the publication of the findings.

## 3 Results

### 3.1 Four individuals with intellectual disability in the family

**FIGURE 1:**
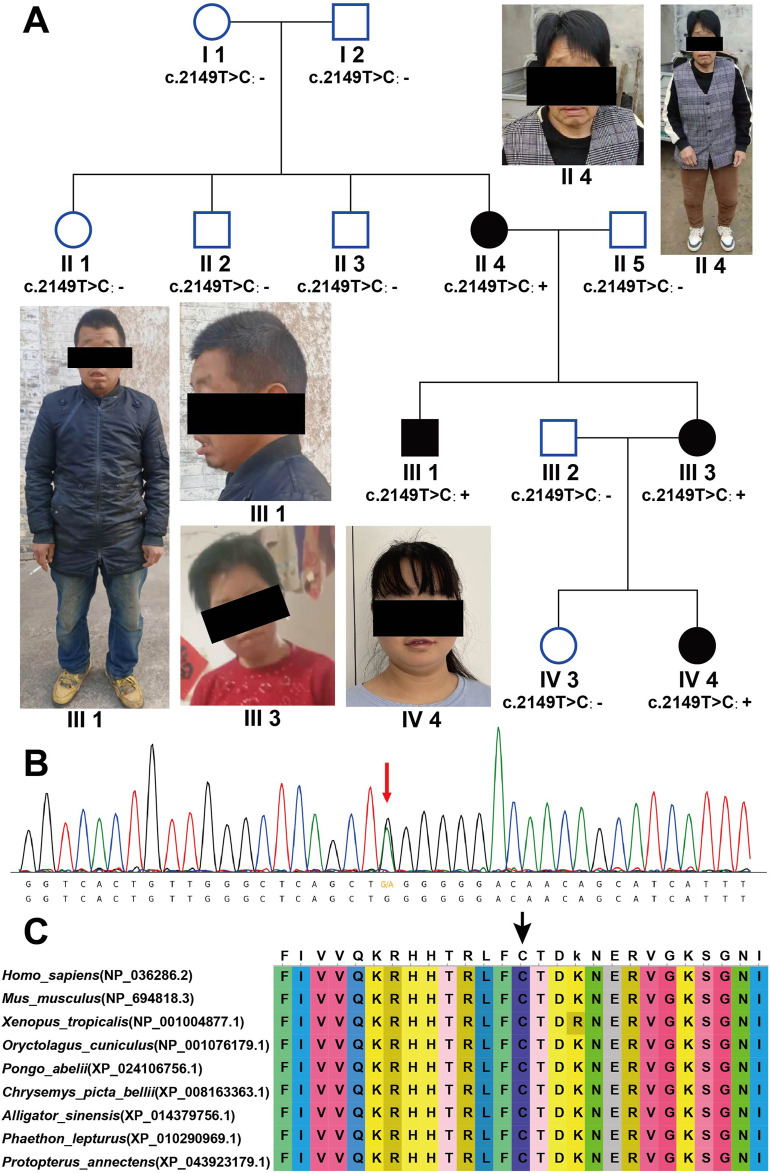
*AGO2* (NM_012154.5): c.2149T>C (p.Cys717Arg) causes LESKRES. **(A)** Pedigree chart of four generations in the LESKRES family. The family includes four LESKRES patients carrying the c.2149T>C (p.Cys717Arg) variant. Healthy family members do not carry this variant. **(B)** Sanger sequencing confirmed that LESKRES patients in this family carry the c.2149T>C (p.Cys717Arg) variant. **(C)** The Cys amino acid at position 717 of the *AGO2* protein is highly conserved across different species.

The proband is a 22-year-old female, with primary symptoms including intellectual disability, motor developmental delay, impaired speech development, impaired receptive language, gait abnormalities, attention deficit hyperactivity disorder, visual impairment, open mouth appearance, low nasal bridge, small nose, and lower eyebrow tail. The family includes four affected individuals: the proband (IV4), along with II4, III1, and III3. The pedigree is shown in Figure 1A. These four affected individuals in the family exhibit similar symptoms. All four affected individuals exhibit comparable clinical features, as detailed in Supplementary Table S1.

### 3.2 *AGO2*: c.2149T>C (p.Cys717Arg) variant co-segregates with intellectual disability

First, whole-exome sequencing (WES) was performed on the proband (IV4) and her parents (III2, III3). The results showed that both the proband (IV4) and her mother (III3) carried the *AGO2* (NM_012154.5): c.2149T>C (p.Cys717Arg) variant, which was confirmed by Sanger sequencing, as shown in Figure 1B. Additional variants identified by Trio-WES included the following: 1) *ALDH18A1* (NM_002860.4): c.683C>T (p.P228L), a *de novo* variant classified as VUS (PM2_P + PP3). 2) *ASH1L* (NM_018489.3): c.6238G>A (p.V2080I), a paternally inherited variant, also classified as VUS (PM2_P + PP3). 3) *TUBGCP2* (NM_006659.4): compound heterozygous variants c.854A>G (p.Y285C), inherited from the mother and classified as VUS (PM2_P + PP3), and c.373G>A (p.A125T), inherited from the father and likewise classified as VUS (PM2_P + PP3). Taking into account the mode of inheritance and the clinical phenotype, we preliminarily consider the *AGO2* (NM_012154.5): c.2149T>C (p.Cys717Arg) variant to be the likely cause of the proband’s intellectual disability. To further confirm the pathogenicity of this variant, peripheral blood was collected from all family members, and Sanger sequencing was used to check whether they carried the above variant. The Sanger sequencing results showed that all the proband (IV4), her mother (III3), her uncle (III1), and her grandmother (II4) carried the variant, while other intellectually normal family members did not carry the variant. The proband’s grandmother (II4) carried a *de novo* variant. The status of symptoms and variant in this family are shown in Figure 1A.

Next, we assessed the pathogenicity of the *AGO2* (NM_012154.5): c.2149T>C (p.Cys717Arg) variant according to the ACMG guidelines. The pathogenicity rating was classified as Likely Pathogenic (PM2_P+PP2+PS2_M+PP1+PP3). The evidence items are as follows: 1) PM2_P: The variant was not found in the gnomAD database. 2) PP2: The missense Z score in the gnomAD database is 6.06, which is greater than 3.09.3) PS2_M: Individual II4 carries a verified *de novo* variant, and her symptoms are consistent with, but not specific to LESKRES syndrome. 4) PP1: The inheritance pattern of *AGO2* is autosomal dominant, with three affected segregations, yielding an LOD score of 0.9, which is greater than 0.6.5) PP3: Multiple prediction tools suggest that this variant is damaging (SIFT, Polyphen2_HVAR, LRT, MutationTaster, PROVEAN, VEST3, M_CAP, CADD, DANN, FATHMM_MKL, Eigen, GenoCanyon, fitCons_pred, ReVe, ClinPred_pred, REVEL). We also analyzed the conservation of the amino acid residue at position 717, and the results showed that the amino acid at position 717 is highly conserved across species (Figure 1C). The amino acid at position 717 is predicted to be conserved by multiple software tools (GERP, phyloP, phastCons, SiPhy_pred).

### 3.3 The p.Cys717Arg variant affect *AGO2* 3D structure

**FIGURE 2:**
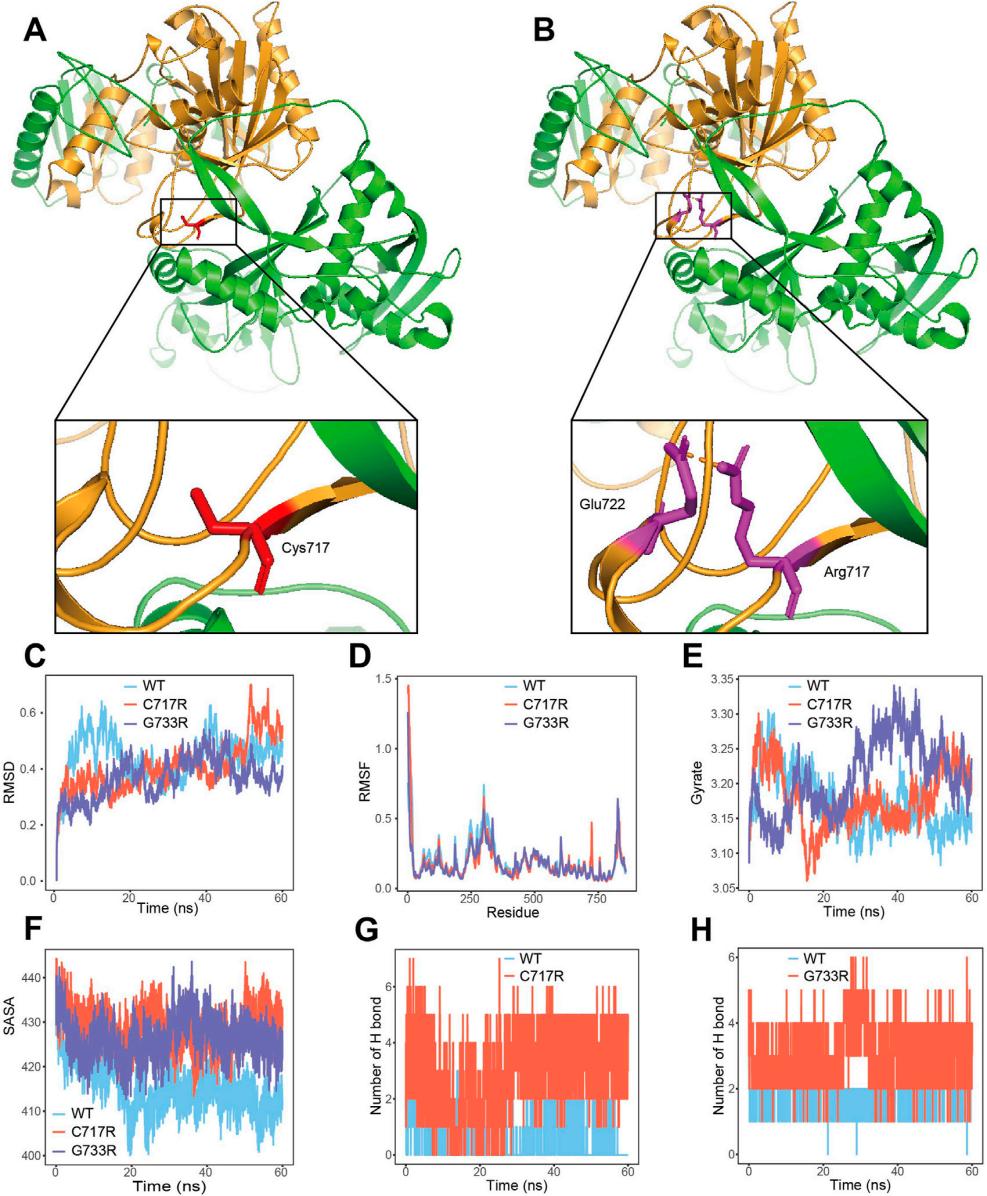
p. Cys717Arg mutant affect *AGO2* 3D structure. **(A)** The 3D structures of wild-type *AGO2* protein. Amino acid residues 517–818 (shown in brown) represent the Piwi domain of the *AGO2* protein. **(B)** The 3D structures of c.2149T>C (p.Cys717Arg) mutant *AGO2* protein. The Piwi domain of the *AGO2* protein are labeled as brown. Hydrogen bond between the Arg 717 and Glu 722 residue are depicted in dotted brown lines. **(C)** The trajectory of RMSD for wide-type (WT) model, p. Cys717Arg (C717R) model and p. Gly733Arg (G733R) model. **(D)** RMSF of the three proteins calculated from each simulation. **(E)** Gyrate of the three proteins calculated from each simulation. **(F)** The p. Cys717Arg and p. Gly733Arg variant enlarges SASA of *AGO2* protein. **(G)** The number of hydrogen bonds between the amino acid at position 717 and other residues in the wide-type and mutant protein. **(H)** The number of hydrogen bonds between the amino acid at position 733 and other residues in the wide-type and mutant protein.

The 3D structures of wild-type *AGO2* protein and the Cys717Arg variant are shown in Figures 2A,B. Amino acid residues 517–818 (shown in brown) represent the Piwi domain of the *AGO2* protein. In the wild-type protein, the Cys residue at position 717 does not form hydrogen bonds with other amino acid residues (Figure 2A). However, in the Cys717Arg variant, the Arg residue at position 717 forms a hydrogen bond with the Glu residue at position 722 (Figure 2B). In the 60 ns molecular dynamics simulation, the Gly733Arg variant was used as a positive control. Compared to the wild-type protein, the number of hydrogen bonds formed between the amino acid residue at position 717 and other residues in the protein was markedly increased in the Cys717Arg variant protein (Figure 2G). Similarly, in the positive control Gly733Arg variant protein, the number of hydrogen bonds formed between the residue at position 733 and other residues within the protein was also significantly increased (Figure 2H). Additionally, the SASA of the Cys717Arg and Gly733Arg variant was significantly greater than that of the wild-type protein (Figure 2F). The RMSD, RMSF and gyrate of the Cys717Arg and Gly733Arg variants showed no significant changes (Figures 2C-E).

### 3.4 Pathogenic *AGO2* gene variants: predominantly missense

**FIGURE 3:**
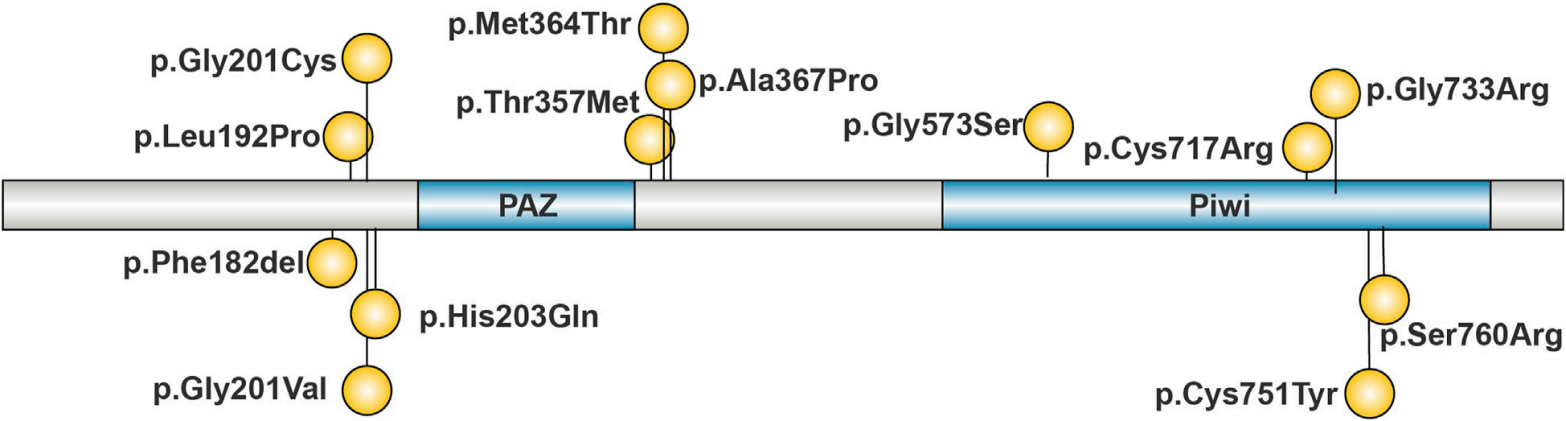
Reported Pathogenic Variants of *AGO2*. Pathogenic variants are concentrated in four regions of *AGO2*: 182–203, 340–367, and 714–760. The majority of these variants are missense mutations.

This study summarized the pathogenic *AGO2* variants reported to date, as illustrated in Figure 3. Including the variant identified in this study, a total of 14 pathogenic variants have been reported. Notably, the chr8.hg19:g. (141,582,269–141,817,600) del variant is not shown in Figure 3. With the exception of p.Phe182del, all identified variants are missense. Analysis of the distribution of pathogenic variants in the *AGO2* gene reveals three hotspot regions at amino acid residues 182–203, 357–367, and 717–760. The p.Cys717Arg variant identified in this study is located within the 717–760 region, which corresponds to the Piwi domain.

Building upon the work of Lessel et al., this study also summarizes the clinical manifestations associated with *AGO2* variants, as detailed in Supplementary Table S1. Phenotypic analysis of affected individuals indicates that intellectual disability (100%, 25/25), motor developmental delay (100%, 25/25), delayed independent walking (100% > 15 months, 22/22), impaired speech development (100%, 25/25), and impaired receptive language (100%, 17/17) are the most frequently clinical features. Seizures (67%, 8/22), epicanthic folds (64%, 16/25), gait abnormalities (59%, 9/18), attention deficit hyperactivity disorder (63%, 12/19), Abnormality of brain morphology (53%, 9/17), and open mouth appearance (52%, 13/25) are the secondly frequently clinical features.

## 4 Discussion

*AGO2* (MIM*606229) variants lead to LESKRES (MIM #619149), an autosomal dominant disorder, with symptoms including global developmental delay, intellectual disability, and delays in speech and language development (Lessel et al., 2020). The symptoms of LESKRES lack specificity compared to other neurological genetic disorders, making it difficult to establish a definitive etiological diagnosis. Genetic testing is necessary to confirm that *AGO2* variations are the cause of LESKRES.

The symptoms observed in the affected individuals reported in this study are consistent with those of LESKRES, with neurological manifestations being the predominant clinical features. Compared to the 21 previously reported cases, the four patients described in this study presented with novel phenotypic features, including low nasal bridge, small nose, and low-set eyebrow tails (Lessel et al., 2020). Regarding congenital anomalies of the skull, the four patients in this study were characterized primarily by a narrow forehead and prominent cheekbones.

In the trio-WES results of the proband and her parents, the *AGO2* (NM_012154.5): c.2149T>C (p.Cys717Arg) variant co-segregated with the proband’s symptoms. The c.2149T>C (p.Cys717Arg) variation is a *de novo* missense mutation, which had not been reported. To better clarify the relationship between c.2149T>C (p.Cys717Arg) variation and the symptoms, we collected peripheral blood samples from family members and used Sanger sequencing to determine whether they carry the variant. The study found that individuals with intellectual disability in the family carried the c.2149T>C (p.Cys717Arg) variant, while normal individuals did not carry this variant. Based on this, we evaluated the variation according to the ACMG guidelines, and classified the variant as Likely Pathogenic (PM2_P+ PP2+ PS2_M+ PP1+PP3).

Among the additional variants related to neurodevelopmental disorders identified by Trio-WES, *ALDH18A1* (NM_002860.4): c.683C>T (p.P228L) and *ASH1L* (NM_018489.3): c.6238G>A (p.V2080I) were *de novo* and paternally inherited, respectively, but did not fulfill the criteria for co-segregation. The inheritance pattern of the compound heterozygous variants in *TUBGCP2* (NM_006659.4): c.854A>G (p.Y285C) and c.373G>A (p.A125T) was also inconsistent with the expected mode of transmission. Therefore, further validation was not performed for these variants.

*AGO2* contains four structural domains: N, PAZ, MID, and Piwi (Nakanishi, 2022). The p. Cys717Arg variant identified in the study is located in the Piwi domain (Figures 2A,B). Several pathogenic/likely pathogenic missense mutations are present near this amino acid position (Figure 3), suggesting that downgrading PM1(PM1_P) could be considered. Since PP2 evidence has already been used, PM1_P evidence was not applied in the pathogenicity classification. The Piwi domain contains a conserved active site aspartate-aspartate-glutamate motif, which has RNA cleavage activity similar to RNAse H, allowing it to cleave substrates (Liu et al., 2004; Song et al., 2004; Parker et al., 2004). In order to determine the effect of the p.Cys717Arg variant on protein function, MD predictive simulation was conducted. The results show that the p. Cys717Arg variant can affect the hydrogen bond formation between the amino acid at position 717 and other amino acids (Figure 2G), thereby affecting the function of the Piwi domain. We hypothesize that the p. Cys717Arg variant affects the *AGO2* protein’s ability to cleave mRNA by influencing the internal hydrogen bonds of the protein. It also affects its interaction with other proteins by altering SASA (Figure 2F), thereby impacting A*GO2* function.

In this study, the pathogenicity of the variant was primarily determined by collecting family information from the patients. This study has several limitations. A lack of sufficient functional experiments to elucidate the precise pathogenic mechanism of the variant represents a major shortcoming. Additionally, standardized clinical evaluations were lacking for some affected individuals within the family.

In summary, this study identified a likely pathogenic novel variant, *AGO2* (NM_012154.5): c.2149T>C (p.Cys717Arg), which causes LESKRES in an autosomal dominant manner. The individuals we reported exhibited previously unrecognized clinical features, thereby enhancing our understanding of the pathogenicity of *AGO2*.

## Data Availability

The datasets presented in this study can be found in online repositories. The names of the repository/repositories and accession number(s) can be found below: https://ngdc.cncb.ac.cn, OMIX008433-01.
